# Heterogeneous Distribution of Genetic Mutations in Myosin Binding Protein-C Paralogs

**DOI:** 10.3389/fgene.2022.896117

**Published:** 2022-06-27

**Authors:** Darshini A. Desai, Vinay J. Rao, Anil G. Jegga, Perundurai S. Dhandapany, Sakthivel Sadayappan

**Affiliations:** ^1^ Department of Internal Medicine, Division of Cardiovascular Health and Disease, University of Cincinnati, Cincinnati, OH, United States; ^2^ Cardiovascular Biology and Disease Theme, Institute for Stem Cell Science and Regenerative Medicine, Bangalore, India; ^3^ Division of Biomedical Informatics, Cincinnati Children’s Hospital Medical Center, Cincinnati, OH, United States; ^4^ Department of Pediatrics, College of Medicine, University of Cincinnati, Cincinnati, OH, United States; ^5^ The Knight Cardiovascular Institute, Oregon Health and Science University, Portland, OR, United States

**Keywords:** *MYBPC1*, *MYBPC2*, *MYBPC3*, hypertrophic cardiomyopathy, distal arthrogryposis

## Abstract

Myosin binding protein-C (MyBP-C) is a sarcomeric protein which regulates the force of contraction in striated muscles. Mutations in the *MYBPC* family of genes, including slow skeletal (*MYBPC1*), fast skeletal (*MYBPC2*) and cardiac (*MYBPC3*), can result in cardiac and skeletal myopathies. Nonetheless, their evolutionary pattern, pathogenicity and impact on MyBP-C protein structure remain to be elucidated. Therefore, the present study aimed to systematically assess the evolutionarily conserved and epigenetic patterns of *MYBPC* family mutations. Leveraging a machine learning (ML) approach, the Genome Aggregation Database (gnomAD) provided variants in *MYBPC1*, *MYBPC2*, and *MYBPC3* genes. This was followed by an analysis with Ensembl’s variant effect predictor (VEP), resulting in the identification of 8,618, 3,871, and 3,071 variants in *MYBPC1*, *MYBPC2*, and *MYBPC3*, respectively. Missense variants comprised 61%–66% of total variants in which the third nucleotide positions in the codons were highly altered. Arginine was the most mutated amino acid, important because most disease-causing mutations in MyBP-C proteins are arginine in origin. Domains C5 and C6 of MyBP-C were found to be hotspots for most mutations in the MyBP-C family of proteins. A high percentage of truncated mutations in cMyBP-C cause cardiomyopathies. Arginine and glutamate were the top hits in fMyBP-C and cMyBP-C*,* respectively, and tryptophan and tyrosine were the most common among the three paralogs changing to premature stop codons and causing protein truncations at the carboxyl terminus. A heterogeneous epigenetic pattern was identified among the three MYBP-C paralogs. Overall, it was shown that databases using computational approaches can facilitate diagnosis and drug discovery to treat muscle disorders caused by *MYBPC* mutations.

## Introduction

Complex diseases stemming from genetic mutations have become a worldwide concern affecting the quality of life. Detecting such genetic diseases depends, in part, on information from online databases (Gudmundsson et al., 2021). Thus, combining such readily accessible data with advanced molecular technologies has helped in identifying various diseases caused by changes in DNA sequences. Indeed, understanding such genetic defects and detecting them at early stages have steadily progressed (Mendez et al., 2021; Yu et al., 2021). No less important is an understanding of conserved elements of the human genome in the context of disease etiology, as well as disease prevention and treatment (Mooney et al., 2010). Researchers now use next-generation sequencing (NGS) methods to determine the order of nucleotides in entire genomes or targeted regions of DNA or RNA, leading to the isolation of genetic mutations likely to develop diseases (Gagan and Van Allen, 2015). Such high-throughput technologies also make it easier to predict the nature of the complex diseases. NGS platforms carry out sequencing of the whole human genome, or a number of small fragments of DNA, at the same time, followed by mapping individual reads to the human reference genome. We can also choose a specific site of interest. Any size of gene can be sequenced to detect the presence of mutations. So far, NGS has successfully identified many disease-causing variants, leading to a better understanding of pathogenic effects and clinical consequences (Schmitt et al., 2012). Moreover, deep machine learning methods can be developed to predict genotype-phenotype outcomes (Al-Numair et al., 2016; de Marvao et al., 2021; Wang et al., 2021).

We herein focused on a group of myosin binding protein-C (MYBPC) paralogs which, together, constitute an immunoglobulin super-family of intracellular muscle proteins (Sadayappan and de Tombe, 2012). MYBPC has three paralogs encoded by unique genes, including slow skeletal (*MYBPC1*), fast skeletal (*MYBPC2*), and cardiac (*MYBPC3*) (Lin et al., 2013). Slow skeletal MyBP-C (sMyBP-C), fast skeletal MyBP-C (fMyBP-C) and cardiac MyBP-C (cMyBP-C) proteins are highly conserved with over 90% homology. They play unique muscle-specific structural and regulatory roles in actomyosin interactions and contractility in striated muscles, including both cardiac and skeletal muscles (Lin et al., 2013). MyBP-C protein is found in the cross-bridge-bearing zone (C region) of A bands in sarcomere of striated muscles (Offer et al., 1973). They provide thick filament stability by interacting with titin and the rod portion of sarcomeric myosin (light meromyosin) through MyBP-C’s C-terminal region (Flashman et al., 2004; Jiang et al., 2015). Ablating *MYBPC2* (Song et al., 2021) and *MYBPC3* (Harris et al., 2002) gene expression results in contractile dysfunction, suggesting the key role played by MyBP-C in striated muscles. It is well known that genetic variants in *MYBPC* genes cause various life-threatening cardiovascular and congenital muscular diseases. For example, mutations in *MYBPC3* are linked to hypertrophic cardiomyopathy (HCM) and dilated cardiomyopathy (DCM) (Bonne et al., 1995; Watkins et al., 1995; Barefield and Sadayappan, 2010; Harris et al., 2011). More than 45% of HCM cases can be attributed to mutations in the *MYBPC3* gene (Spirito et al., 1997). Strikingly, 70% of genetic variants in *MYBPC3* are nonsense mutations, including indels, frameshift, and splice-site mutations, leading to cMyBP-C truncations at the carboxyl terminus (Richard et al., 2010; Harris et al., 2011). It is, however, unclear why *MYBPC3* variants predominantly result in protein truncations and whether any evolutionary reasons behind such preferential variants. In contrast, few variants have been reported in the skeletal paralogs (Desai et al., 2020). However, some recent studies suggest that mutations in *MYBPC1* are linked to a congenital disease called distal arthrogryposis (Bamshad et al., 2009; Stavusis et al., 2019; Desai et al., 2020) and myogenic tremor (Geist Hauserman et al., 2021). Specifically, infants born with *MYBPC1* variants developed with multiple joint contractures congenitally limiting muscular movement and affecting the quality of life (Bamshad et al., 2009; Markus et al., 2012). On the other hand, *MYBPC2* has also been linked to skeletal muscular disorders like arthrogryposis (Bayram et al., 2016). Thus, it is well worth systematically determining the genetic variability among these three genes, such as frequency, a hot spot, differences in codon usage, and degree of pathogenicity.

To date, around 2,000 variants have been reported in the *MYBPC3* gene (Helms et al., 2020), but the conserved pattern and biochemical characteristics of these variants have not been systematically reviewed. Therefore, in the present study, we analyzed variants of all three *MYBPC* gene isoforms for their effects, using the Variant Effect Predictor (VEP) to understand MyBP-C biology and evolutionary pattern (McLaren et al., 2016). The Genome Aggregation Database (GnomAD) (Gudmundsson et al., 2021) was used to collect the up-to-date *MYBPC* variants. We then performed data mining, queried the database of variants reported in these three gene isoforms, and carried out a comprehensive bioinformatics review of the evolutionary pattern of conserved variants in the *MYBPC* gene family. Variants and the similarities among them were compared among the three *MYBPC* paralogs, along with heterogeneous, gene-specific epigenetic patterns.

## Materials and Methods

### Accessing Variant Database and Data Extraction

Variant data for *MYBPC1*, *MYBPC2*, and *MYBPC3* genes were directly downloaded from the Genome Aggregation Database (gnomAD) (Karczewski et al., 2020). This database is open source, and it aggregates and harmonizes exome and genome sequencing data from multiple large-scale sequencing projects. We also used Ensembl’s Variant Effect Predictor (VEP) (McLaren et al., 2016) to obtain annotations for all gnomAD variants from these three genes with an rsID. The collection of variants was genome-wide, including both coding and noncoding regions. We understand that homopolymeric regions pertaining to mitochondrial DNA (mtDNA) variants have been filtered out of gnomAD data, and we carried out our analyses accordingly.

### Variant Identification and Analyses

Analysis was carried out using in-house scripts. The data were first processed by removing any duplicate variant entries. The longest isoforms of *MYBPC1* (transcript ID ENST00000361466), *MYBPC2* (transcript ID ENST00000357701), and *MYBPC3* (transcript ID ENST0000545968) were identified from the resulting files. Variants impacting other genes could then be removed (*AC117505.1* residing within *MYBPC1*; *AC020909.1* and *SPIB*, both downstream of *MYBPC2*, and *FAM71E1* upstream of *MYBPC2*; *MADD* downstream of *MYBPC3* and *SPI1* upstream of *MYBPC3*) (Supplementary Figure S1). Based on the resultant data, we wanted to discriminate among the variations observed across the three genes. First, we identified the frequency of each variant by category, including synonymous, missense, truncation, frameshift, and non-frameshift, indels and others, such as splice donors and acceptors, loss of start and stop codon, and protein-altering variants. Next, we identified the mutated nucleotides in each variant category, as well as codon position of nucleotides mutated in missense and synonymous variants. We also studied protein variants, classifying the amino acid (aa) variations in protein domains, the information of which was obtained through UniProt. Based on nucleotide data obtained earlier, we also investigated the proneness of certain exons to mutations. Last, with the help of variant consequence predictors, such as SIFT and Polyphen, we identified the distribution of pathogenic variants among the three genes (Supplementary Figure S2). Scripts will be provided upon request to the corresponding author.

## Results

### Variable Distribution of Genetic Variants in Myosin Binding Protein-C Paralogs

**FIGURE 1:**
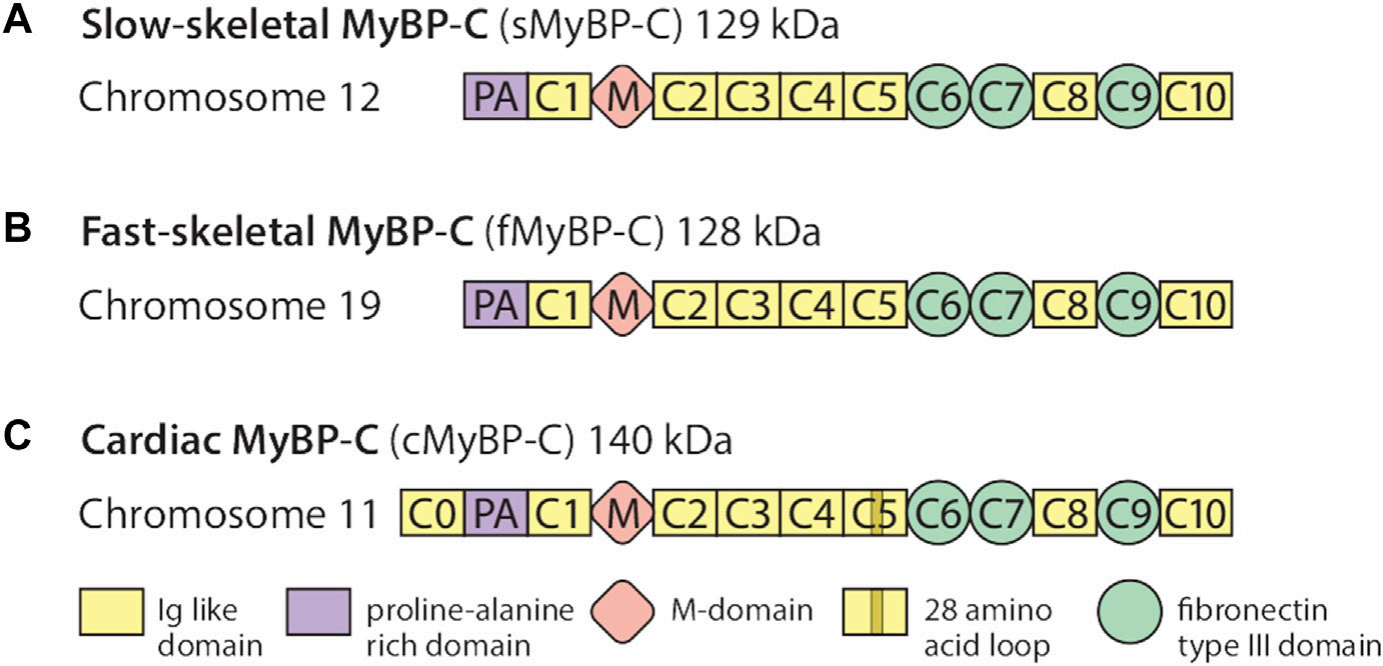
The MyBP-C family consists of one cardiac and two skeletal paralogs. sMyBP-C is a 129 kDa protein encoded by the *MYBPC1* gene contained in chromosome 12 **(A)**, and fMyBP-C is a skeletal muscle-specific protein encoded by the *MYBPC2* gene contained in chromosome 19 **(B)**. The two skeletal paralogs share similar domains, from C1 to C10, which contain three fibronectin type III domains (C6, C7, and C9) and seven immunoglobulin-like domains with one Proline-Alanine (PA)-rich domain and phosphorylation (M) domains. The 140 kDa cMyBP-C is encoded by the *MYBPC3* gene **(C)** and also shares structural features similar to those of the skeletal paralogs, except it has one additional domain in its N′-region (C0). It also has a 28-amino acid loop in its C5 domain.

**FIGURE 2:**
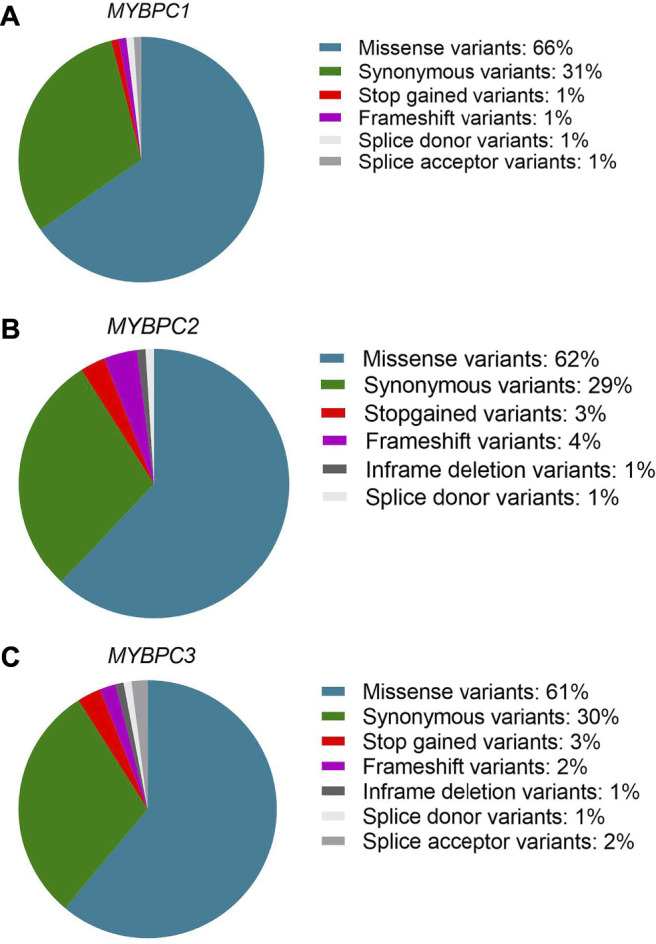
Missense variants predominate across paralogs. Variant ensemble predictor (VEP) analysis shows that the *MYBPC* family shares a similar pattern of variant distribution, predominantly comprised of single nucleotide variants (61%–66% missense variants, 30% synonymous variants). The remainder includes stop-gain, splicing variants, non-frameshift variants and frameshift variants in *MYBPC1* **(A)**, *MYBPC2* **(B)**, and *MYBPC3* **(C)** genes.

**FIGURE 3:**
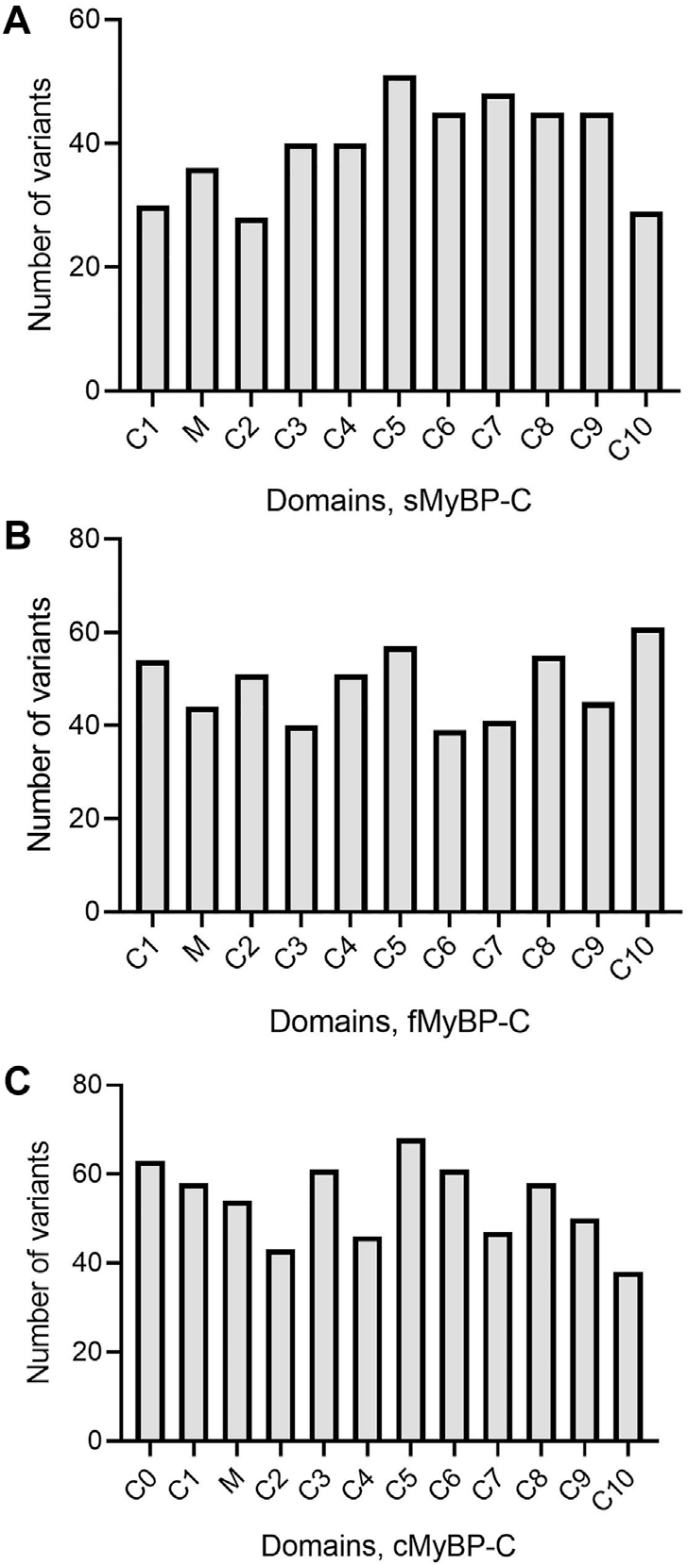
Variant frequency is characterized by heterogeneity in *MyBP-C paralogs*. Heterogeneous distribution of variant frequency observed in the domains of the MYBPC family. **(A–C)** sMyBP-C has the lowest number of variants across domains, compared to other paralogs, and C5 domain was the most mutated domain with the highest number of variants in the MYBPC family.

The MyBP-C protein family is a group of thick filament accessory proteins regulating striated muscle structure and function. cMyBP-C differs from sMyBP-C and fMyBP-C proteins by containing a unique C0 domain and 28 aa loops at the C5 domain (Figure 1). sMyBP-C is highly homologous to fMyBP-C, but its expression and functions differ (Dhoot et al., 1985; Weber et al., 1993). To determine the conserved pattern in MyBP-C structural biology, we analyzed 8,617, 3,870, and 3,070 variants in *MYBPC1*, *MYBPC2*, and *MYBPC3* genes, respectively. Variants were collected from GnomAD, and annotations were calculated by VEP. Among coding variants across the three paralogs, VEP analysis revealed missense variants to be the most predominant (61%–66%), followed by synonymous variants (∼30%), frameshift and truncation variants (∼3%), and then in-frame indels and splice-site variants (2%–3%) (Figure 2). While *MYBPC3* had the highest number of coding variants (Supplementary Figure S2), *MYBPC1* had the highest number of variants with intronic variants, making up 90% of variants in *MYBPC1* as compared to 75% in *MYBPC2* and 62% in *MYBPC3* (data not shown). Next, we analyzed the domain-wise frequency in all MyBP-C proteins (Figure 3). Interestingly, the C5 domain proved to be the most prone to mutations among all three proteins, despite not being the longest domain in sMyBP-C and fMyBP-C (Figures 3A–C).

### Paralog-Specific Alterations in Amino Acids

**FIGURE 4:**
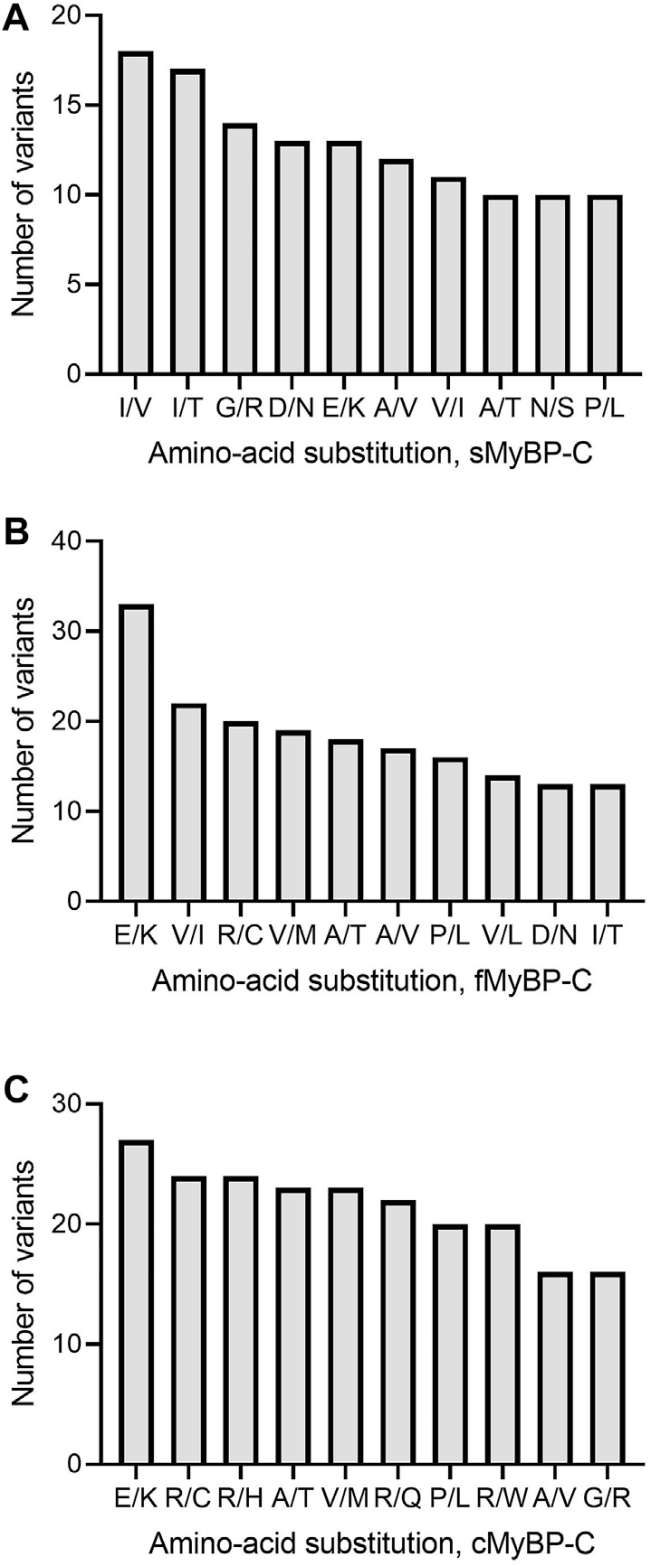
Top ten most frequent amino acid substitutions in the MyBP-C paralogs. E > K and A > T are the most common amino acid substitutions across paralogs. I/V, V/I and R/C are most frequent amino acid substitutions in sMyBP-C **(A)**, fMyBP-C **(B)** and cMyBP-C **(C)**, respectively.

Next, variants were categorized into missense, frameshift, and truncation mutants based on variations in their amino acids. Our analyses revealed that Glu > Lys (E > K) and Ala > Thr (A > T) were the most frequent missense amino acid substitutions across the MyBP-C proteins and that Ile > Val (I > V) was the most frequent in sMyBP-C (Figures 4A–C). Interestingly, among the ten most frequent amino acid substitutions among the three paralogs, there lacks a mutation of the commonly post-translationally modifiable residues (Lys, Ser, Thr, Tyr), except for Arg, however we do commonly observe modifiable amino acids occurring in these proteins as a result of mutations. This feature could be explored as a potential therapeutic target since post-translational modifications are known to frequently activate or de-activate proteins.

**FIGURE 5:**
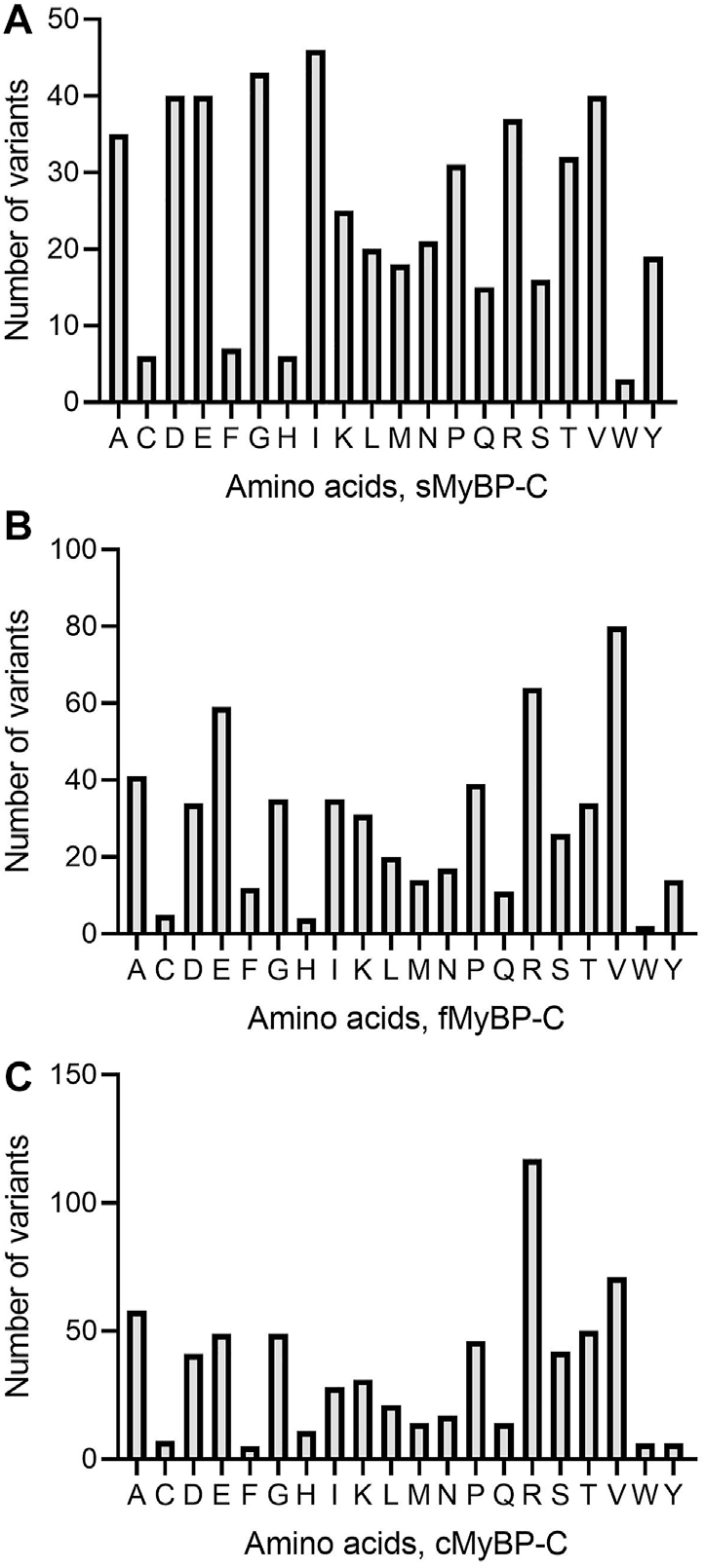
Prevalence of amino acids in missense variants across paralogs. Arg (R) was the most mutated amino acid in missense variants in the MyBP-C protein family with Val (V) being the second most mutated in sMyBP-C **(A)**, fMyBP-C **(B)** and cMyBP-C **(C)**. Iso (I) was another highly mutated amino acid in missense variants in the cMyBP-C protein.

**FIGURE 6:**
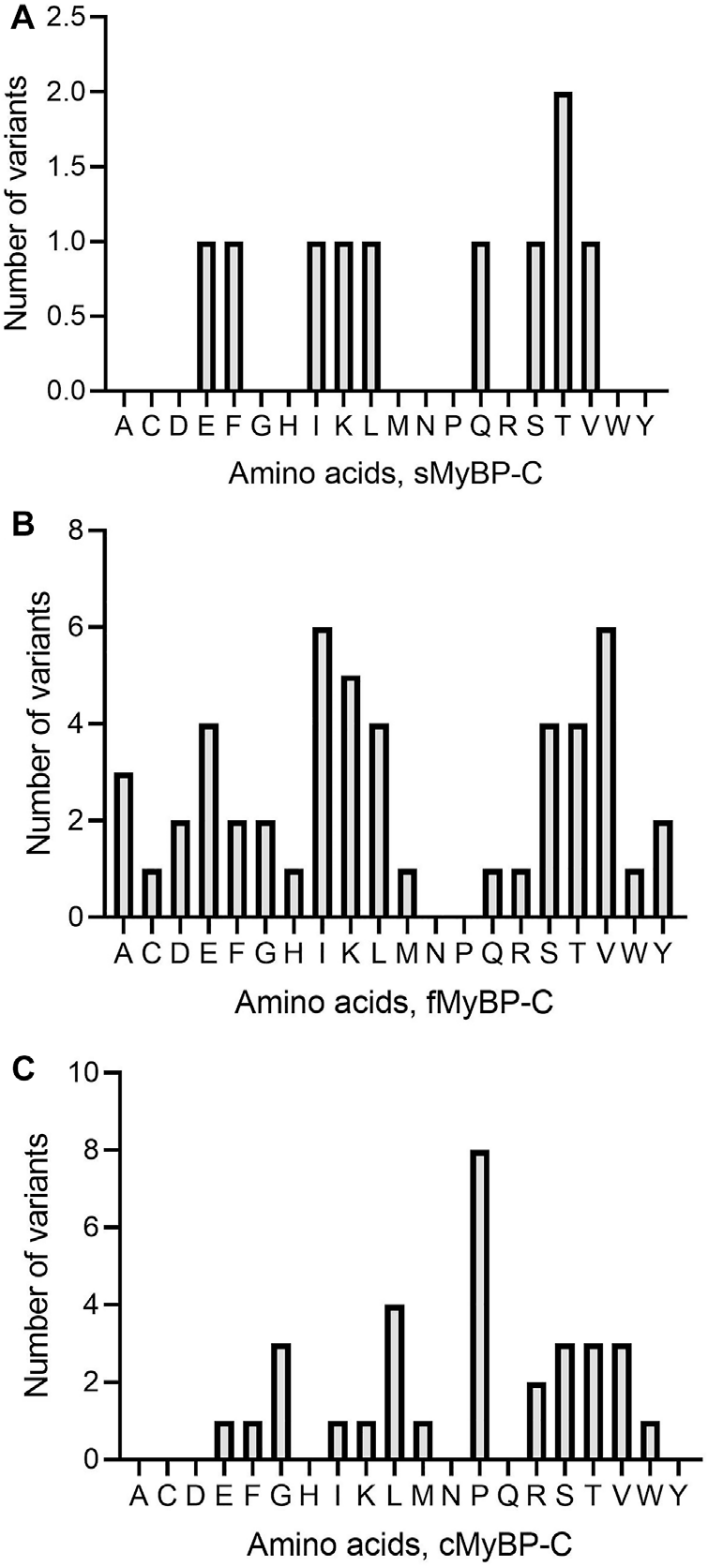
Prevalence of amino acids in frameshift variants in the MyBP-C gene family. Thr (T), Iso (I) and Pro (P) were the most mutated amino acids in frameshift variants in sMyBP-C **(A)**, fMyBP-C **(B)** and cMyBP-C **(C)** proteins, respectively.

Among missense variants, Cys, Phe, His, and Trp were the least mutated amino acids, while, again, Arg and Val were the most frequently mutated amino acids across all *MYBPC* genes (Figures 5A–C). sMyBP-C was shown to have other frequently mutated amino acids, including Ile, Gly, Val, Asp, Ala, and Glu, while fMyBP-C had frequent mutations in Pro, Glu and Val, and cMyBP-C showed no affinity towards mutations in any amino acids other than Val and Arg. This could be attributed to an excess number of codons coding for Arg. However, this pattern dramatically changed in frameshift variants with most mutations impacting Thr (sMyBP-C), Ile (fMyBP-C), and Pro (cMyBP-C), respectively (Figures 6A–C). Arg was largely unaffected by frameshift mutations.

**FIGURE 7:**
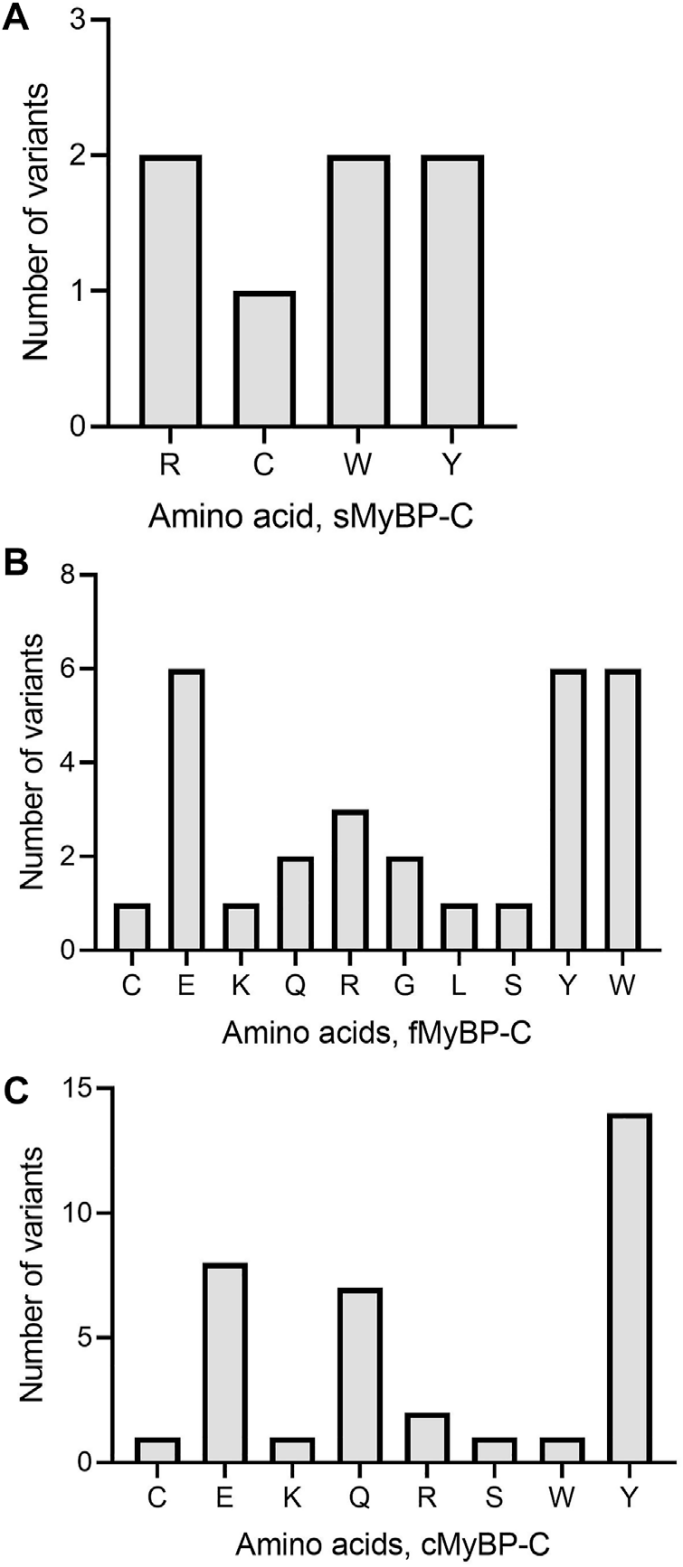
Prevalence of amino acids targeted to introduce premature termination in the MyBP-C gene family. Trp (W) and Arg (R) were the top amino acids introducing a prematurely terminated stop codon in sMyBP-C protein **(A)**, and Glu (E) was the most targeted amino acid in both fMyBP-C **(B)** and cMyBP-C **(C)** protein. Lastly, Tyr (Y) was the most prevalent amino acid leading to premature termination and causing C′-terminal truncation in all three paralogs.

Another important category of mutation includes truncation variants since they might not include regulatory or functional domains in the translated protein. In the MyBP-C family, not surprisingly, we mostly observe Trp and Tyr mutations leading to the introduction of a premature stop codon (Figures 7A–C). In sMyBP-C, however, Arg variants leading to truncated variants are as common as Trp and Tyr (Figure 7A). Glu mutations leading to truncation could be observed in both fMyBP-C and cMyBP-C (Figure 7).

### Variant Distribution Across Exons and Domains in the Paralogs

**FIGURE 8:**
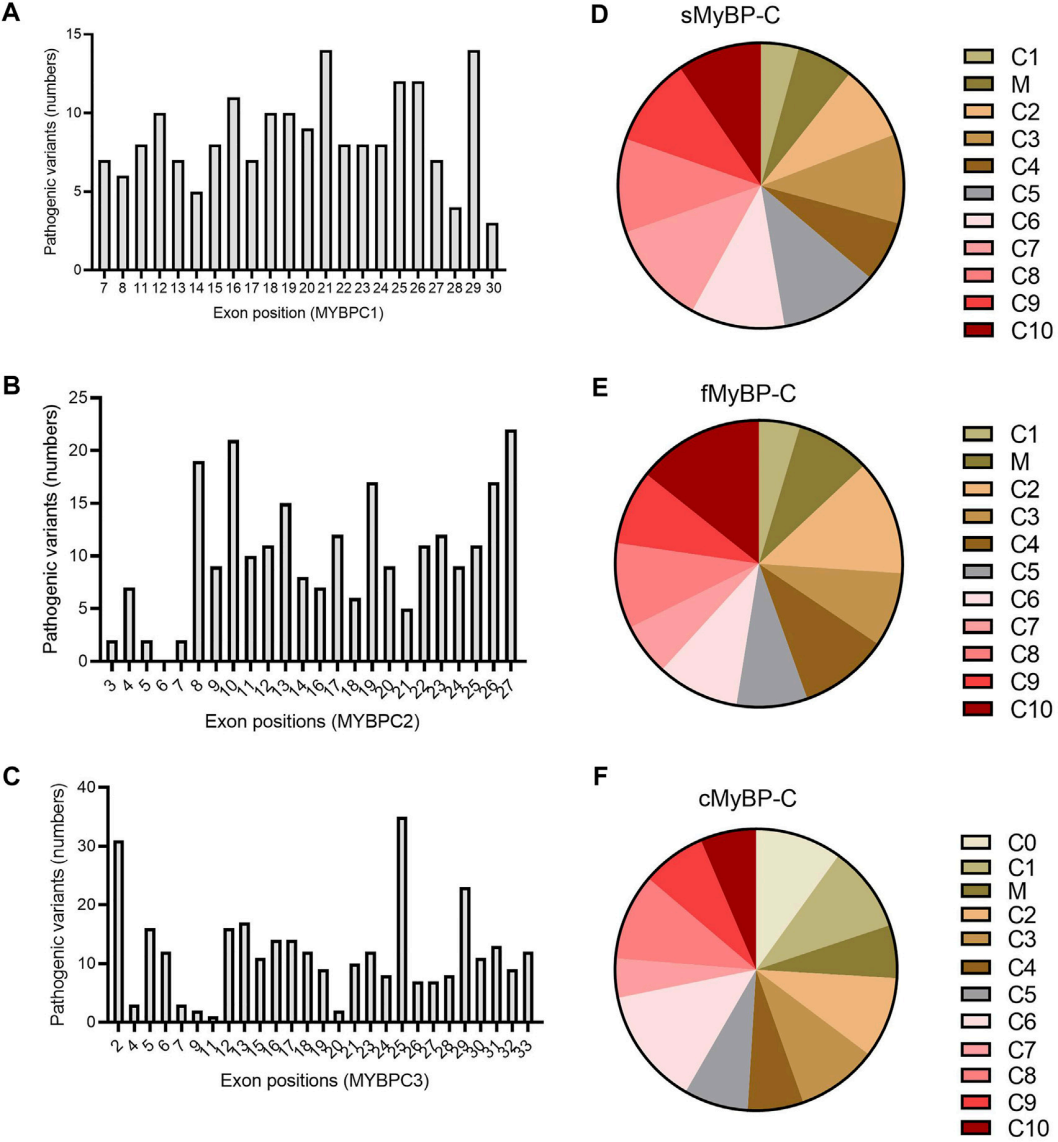
Prevalence of exons in the genes and domains of proteins susceptible to pathogenic variants in *MYBPC* gene paralogs. In *MYBPC1* **(A)** *,* exons 21 and 29 had the highest number of hits with pathogenic variants. However, in *MYBPC2* **(B)**, exons 8, 10, 19, 26, and 27 were the prevalent targets with pathogenic variants. In *MYBPC3* **(C)**, exon 2 and exon 25 had the highest number of variants. In terms of domain-wise frequency of pathogenic variants, the C6 domain was the most highly mutated domain leading to pathogenic variants across paralogs, followed by C5 and C7 in sMyBP-C **(D)**, C2 and C10 in fMyBP-C **(E)** and C0, C1, and C6 in cMyBP-C **(F)** proteins.

Next, we applied filters to the VEP files in order to categorize variants as “likely pathogenic” and analyzed which domains and exons were the most susceptible to mutation. Again, all three paralogs showed very heterogeneous distribution in the frequency of pathogenic variants (Figures 8D–F). However, while mutations in the C10 domain caused pathogenic variants in fMyBP-C, the C10 domain of cMyBP-C was the least mutated. Instead, C6 was the most mutated domain. Heterogeneous distribution characterized sMyBP-C in which domains comprising the N terminal of the protein were found to be the least mutated. Next, we investigated which exons contained the most pathogenic variants. Here, although *MYBPC1* showed very mixed distribution, exons 21 and 29 contained most pathogenic variants. Exons 8, 10, 26, and 27 contained the most pathogenic variants for *MYBPC2*, whereas exon 25 was clearly the most pathogenic variant-containing exon for *MYBPC3*, followed by exons 2 and 29. (Figures 8A–C). Distal Arthrogryposis has been attributed to pathogenic variants in *MYBPC1*, and *MYBPC3* is known to cause a series of cardiomyopathies, including HCM, DCM and congenital heart defects. Mutations in *MYBPC2* were not very well annotated in terms specific diseases, but a few variants were linked to cognitive dysfunction, according to the VEP files.

## Discussion

MYBPC paralogs play a major role in striated muscle contraction. Increasing evidence suggests that genetic alterations in *MYBPC* paralogs are directly linked to myopathies. However, no systematic analyses have been carried out to determine nucleotide pattern, codon, and amino acid changes in existing genetic mutations among these three proteins. We wanted to understand the patterns of genetic variants arising from evolutionarily conserved amino acids in MyBP-C structural biology. To this end, we analyzed around 3,000 variants in each paralog obtained from GnomAD and calculated the annotations using the variant effect predictor (VEP). The collected data were analyzed by comparing all three paralogs. Mapping variant frequency in the domains of all MyBP-C proteins revealed a heterogeneous distribution, indicating that all domains in the MyBP-C protein are equally susceptible to mutation. Very few studies have reported on the conserved sequences among the three MYBPC paralogs (Okagaki et al., 1993; Weber et al., 1993; Shaffer and Gillis, 2010; Lin et al., 2013). However, in the present study, the conserved pattern of *MYBPC* mutations also showed a very heterogeneous distribution in all three paralogs. Missense variants predominated with Ile as the most mutated amino acid in sMyBP-C, Val in fMBP-C and Arg in cMyBP-C.

(Shaffer and Gillis, 2010) reported a high level of conserved sequences. The M-domain, otherwise known as the MyBP-C motif, contains a unique set of 100 amino acids at the N terminus between domains C1 and C2. This region is essential for actomyosin interactions. The M-domain binds myosin S2, as well as actin, to regulate cross-bridge formation during contraction and relaxation. Upon phosphorylation by kinases like PKA, the bond between M-domain and S2, or actin, is broken, allowing cross-bridge formation (Gruen et al., 1999; Korte et al., 2003; Stelzer et al., 2007; Shaffer et al., 2009). Many regions of the M domain are highly conserved, including 293–300 and 331–353 in humans, which may, or may not, carry functional importance. However, some regions of MyBP-C are not well conserved and are unique to the cardiac paralog. The cardiac cMyBP-C isoforms contain an additional ∼100 amino acid domain at the extreme N terminus called the C0 domain which is absent in the slow and fast skeletal paralogs (Figure 1). In the same evolutionary study by Shaffer and Gillis, the phylogenetic analysis of MyBP-C sequences revealed MyBP-C paralogs to be monophyletic, while the fast and slow skeletal MyBP-C protein paralogs clustered in a group that deviated from that of cMyBP-C. This indicated that cMyBP-C is the ancestral form of MyBP-C. They also predicted that gene duplication events caused changes in the sequence of cMyBP-C, resulting in the differentiation of slow skeletal from the cardiac paralog. Differences in the sequence of cMyBP-C enable it to carry out its specialized cardiac muscle function (Shaffer and Gillis, 2010).

Previous alignment studies noted a significant degree of conserved sequences across all three paralogs (Shaffer and Gillis, 2010). A high number of amino acids were found to be conserved in MyBP-C, depending on the species. Altogether, eight residues in mammalian cMyBP-C switch from nonpolar amino acids to charged amino acids in the other two isoforms (Shaffer and Gillis, 2010). For example, in human cMyBP-C, as well as all other mammalian cardiac isoforms, Gly-354 (or its equivalent) can be observed, while an Arg residue can be found at the equivalent site in nonmammalian cardiac isoforms, as well as all fast and slow skeletal isoforms. The functional importance of these amino acids is unknown (Shaffer and Gillis, 2010). This could explain why Arg was the most mutated amino acid in cMyBP-C and the other paralogs.

Life-threatening diseases have been attributed to mutations in the MYBPC paralog proteins. For example, mutations in the *MYBPC3* gene are also linked to HCM, DCM and sudden cardiac death. Missense mutations cause stable proteins to incorporate into the sarcomere and lead to various functional defects. However, frameshift mutations result in a prematurely terminated codon in the transcribed mRNA, making C-terminal truncated proteins unable to bind myosin or titin and also leading to functional defects. About 70% of genetic variants in *MYBPC3* represent C′’-truncations (Harris et al., 2011). Furthermore, C-terminal truncated proteins have never been detected by immunoblots in cardiac tissue of HCM patients (Marston et al., 2009; van Dijk et al., 2009). Importantly, cardiomyocytes from cMyBP-C mutants are markedly decreased from those of the wild-type protein (Yang et al., 1999). Altogether, these studies strongly suggest that mutant proteins and/or mRNAs are unstable and degraded accordingly. Therefore, it was proposed that frameshift and nonsense mutations might lead to cMyBP-C haploinsufficiency (van Dijk et al., 2009; Suay-Corredera et al., 2021). A non-functional or mutant protein incorporating into the sarcomere can cause filament disassembly, altered function, and, finally, HCM- or DCM-like phenotype (Schlossarek et al., 2012). While few studies have reported on *MYBPC1* and *MYBPC2*, many studies have linked *MYBPC1* mutations to distal arthrogryposis (Desai et al., 2020). For example, one study found that *MYBPC1* mutations W236R and Y856H could cause distal arthrogryposis type 1 (Gurnett et al., 2010). Another study in a Chinese family found that E359K, R318X, and P319L mutations led to distal arthrogryposis type 2 (Li et al., 2015). Distal arthrogryposis is a skeletal muscle disorder characterized by joint contractures and deformities on distal body parts immobilizing muscle movements (Desai et al., 2020). Few *MYBPC2* mutations have been explained clinically. However, a recent study from our laboratory shows that global knockout of *MYBPC2* in mice results in reduced contractility and reduced myofilament calcium sensitivity and hypertrophic response to mechanical overload (Song et al., 2021).

Some disease-causing founder mutations are specific to ethnicity and limited to a geographic region (Dhandapany et al., 2009). In recent years, *MYBPC3* has gained significant interest owing to its role in the regulation of contractility in the sarcomere machinery. *MYBPC3* is known to regulate contraction upon its phosphorylation by various kinases (van Dijk et al., 2009). Studies have shown that C′-truncation of *MYBPC3* causes cMyBP-C null and DCM in mice at the age of 3 months (McConnell et al., 1999), as well as significant epigenetic changes (Tabish et al., 2019), indicating the importance of normal cMyBP-C for regular cardiac function (Lynch et al., 2015). In comparison, much less is known about the two skeletal isoforms of MyBP-C (McNamara and Sadayappan, 2018).

Across the three genes, we observed the highest prevalence of coding variants and pathogenic coding variants in *MYBPC3*. While missense variants constitute most of the coding variants, a considerable prevalence of loss-of-function variants can be seen in the three genes represented by frameshift and truncation variants (Figure 2). From an evolutionary perspective, we also noted in all genes that the C5 domain is highly prone to variants, which could, therefore, be a potential therapeutic target in disease conditions. In *MYBPC3*, the cardiac specific N-terminal domain is also highly prone to variations, thus possibly crucial in the treatment of HCM (Figure 3). A stark change in the polarity of amino acids is observed in missense mutations, potentially altering protein binding and/or key post-translational modifications in MyBP-C proteins, thereby leading to a disease phenotype (Figure 4).

In our analyses, the most dominant variant among the MyBP-C paralogs was missense variants, followed by frameshift variants. For instance, a missense variant, like R403Q or R663H, ablates the binding of myosin with the C0-C7 fragment of cMyBP-C protein and causes hypercontractility (Sarkar et al., 2020). The N-terminal of cMyBP-C regulates contractility within the sarcomere machinery. Mutations in the domains comprising the N-terminal often lead to cardiac dysfunction. Our analysis revealed the C6 domain of cMyBP-C to have the most pathogenic variants, followed by C0 and C1. Nearly half of these variants localize to the C0-C4, comprising the N terminal of the protein. Mutations in the N -terminal can lead to either reduced or increased binding with the myosin region, depending on the mutation. This explains the loss- or gain-of-function in the case of mutations leading to cardiac dysfunction. As mentioned previously, MyBP-C has many conserved domains, and, over time, mutations can negatively impact contractile function. In our study, Glu > Lys (E/K) was most frequent amino acid substitution found in all MyBP-C paralogs. A switch from a negatively charged amino acid to a positively charged amino acid can lead to loss of binding to the neighboring amino acids in a protein and ultimately to reduced or increased function in the sarcomere. Arg was the most mutated amino acid across all three paralogs of MyBP-C missense variants. However, Arg was also the least susceptible to frameshift mutation, suggesting that the nucleotides coding for Arg do not often change the entire sequence of amino acids in the protein, another avenue for exploration in further studies. Among the pathogenic variants, exon 25 and 29 of *MYBPC1* and *MYBPC3* were the most likely to be mutated, suggesting the likelihood that these positions can destabilize the structure in a manner sufficient to alter the protein’s functionality. However, for *MYBPC2,* mutations in the exons 8, 10, and 27 were most likely associate with distal arthrogryposis (Desai et al., 2020).

In conclusion, our study demonstrates the evolutionary pattern of conserved variants in the MyBP-C family of proteins, potentially leading to complex genetic diseases. Overall, the results of our assessment can be used for genetic mapping and identifying genetic variants in individuals with a history of such mutations for the purpose of clinical diagnosis and prognosis.

## Limitations of the Study

In this study, only the variants available on the gnomAD database were analyzed. Other variants may be present in other databases for *MYBPC* genes. Since the data in gnomAD represent aggregate data, phenotype and other individual-level data are not available. Follow-up studies can be undertaken using data from biobanks such as the UK Biobank. Additionally, gnomAD has an over-representation of data from European populations compared to participants from other communities (e.g., Middle Eastern, and Oceanian populations. Despite rigorous quality control, gnomAD may also contain sequencing and annotation artifacts (Gudmundsson et al., 2021). We used only one effect predictor, namely VEP, to annotate the variants. Current variant annotation tools, including VEP, annotate each variant independently and do not consider the potential compound effects of combining alternate alleles. In other words, two or more variants affecting the same codon are not considered when annotating. While VEP, or similar predictors, can predict the functional effects of genomic variants, without validation studies, the predicted deleterious variants cannot be claimed as absolutely pathogenic or cause a definite phenotype. With subjects’ samples (control and case datasets) underlying mechanisms of pathogenesis caused by these variants can be deduced.

## Data Availability

The original contributions presented in the study are included in the article/Supplementary Material, further inquiries can be directed to the corresponding author.

## Glossary

cMyBP-C: Cardiac myosin binding protein-C protein
fMyBP-C: Fast skeletal myosin binding protein-C protein
sMyBP-C: Slow skeletal myosin binding protein-C protein
MyBP-C: Myosin binding protein-C protein
MYBPC1: Slow skeletal myosin binding protein-C gene
MYBPC2: Fast skeletal myosin binding protein-C gene
MYBPC3: Cardiac myosin binding protein-C gene
Ala (A): Alanine
Arg (R): Arginine
Asn (N): Asparagine
Asp (D): Aspartic acid
Cys (C): Cysteine
DCM: Dilated cardiomyopathy
Gln (Q): Glutamine
Glu (E): Glutamic acid
Gly (G): Glycine
His (H): Histidine
HCM: Hypertrophic cardiomyopathy
Ile (I): Isoleucine
Leu (L): Leucine
Lys (K): Lysine
Met (M): Methionine
Phe (F): Phenylalanine
Pro (P): Proline
Pyl (O): Pyrrolysine
Ser (S): Serine
Thr (T): Threonine
Trp (W): Tryptophan
Tyr (Y): Tyrosine
Val (V): Valine
**SNP**: Single nucleotide polymorphism
NGS: Next-generation sequencing
