# Supplementary material for: Heterogeneous Distribution of Genetic Mutations in Myosin Binding Protein-C Paralogs
Source: Front Genet. 2022 Jun 27;13:896117. doi: 10.3389/fgene.2022.896117 (PMC9272480; doi:10.3389/fgene.2022.896117)
Supplement: Supplementary file 1 [file DataSheet1.PDF]

SUPPLEMENTAL DATA

Original Article – Frontiers in Genetics

Heterogeneous distribution of genetic mutations in myosin binding protein-C paralogs

Darshini Desai[a*], Vinay J. Rao[b*], Anil G. Jegga[c], Perundurai S. Dhandapany[b,d] and Sakthivel Sadayappan[a]

a) Department of Internal Medicine, Division of Cardiovascular Health and Disease, University of Cincinnati, Cincinnati OH 45267, USA.
b) Centre for Cardiovascular Biology and Disease, Institute for Stem Cell Biology and Regenerative Medicine, Bangalore, India.
c) Division of Biomedical Informatics, Cincinnati Children's Hospital Medical Center, 240 Albert Sabin Way, MLC 7024, Cincinnati, OH 45229, USA.
d) The Knight Cardiovascular Institute, Oregon Health and Science University, Portland, OR, USA.

*These two authors contributed equally to this paper.

Short-title: Genetic variants in myosin binding protein-C paralogs

Correspondence to Sakthivel Sadayappan, PhD, MBA, Division of Cardiovascular Health and Disease, University of Cincinnati, 231 Albert Sabin Way, Cincinnati, OH 45267, USA. Phone: +1 513 558 7498; Email: sadayasl@ucmail.uc.edu

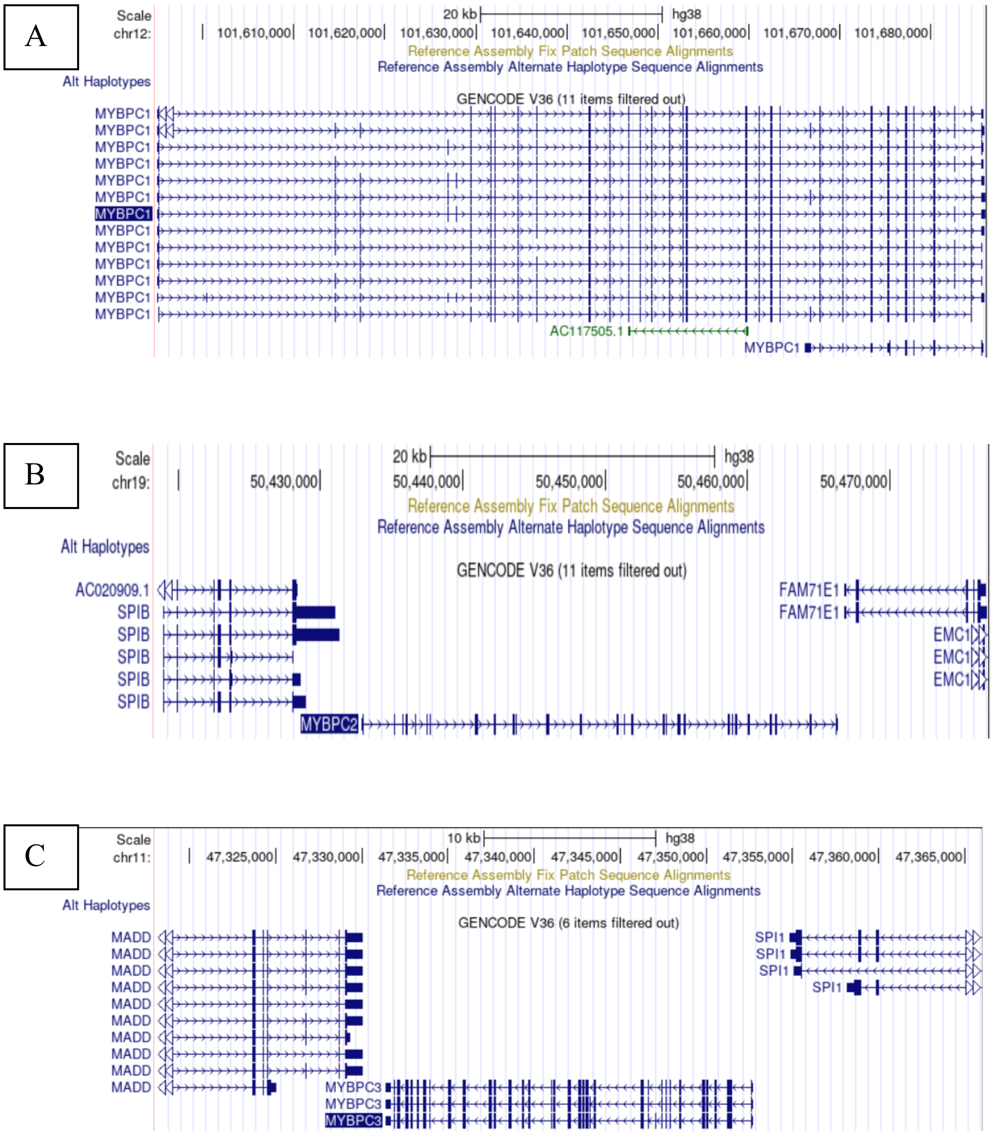

Supplementary Figure 1: UCSC genome browser views of the three *MYBPC* genes. Blue represents coding genes, and green represents non-coding genes. A, *MYBPC1* (on the + strand) and AC117505.1. B, *MYBPC2* (on the + strand) flanked by AC020909.1, FAM71E1 and SPIB. C, *MYBPC3* (on the – strand) flanked by SPI1 and MADD.

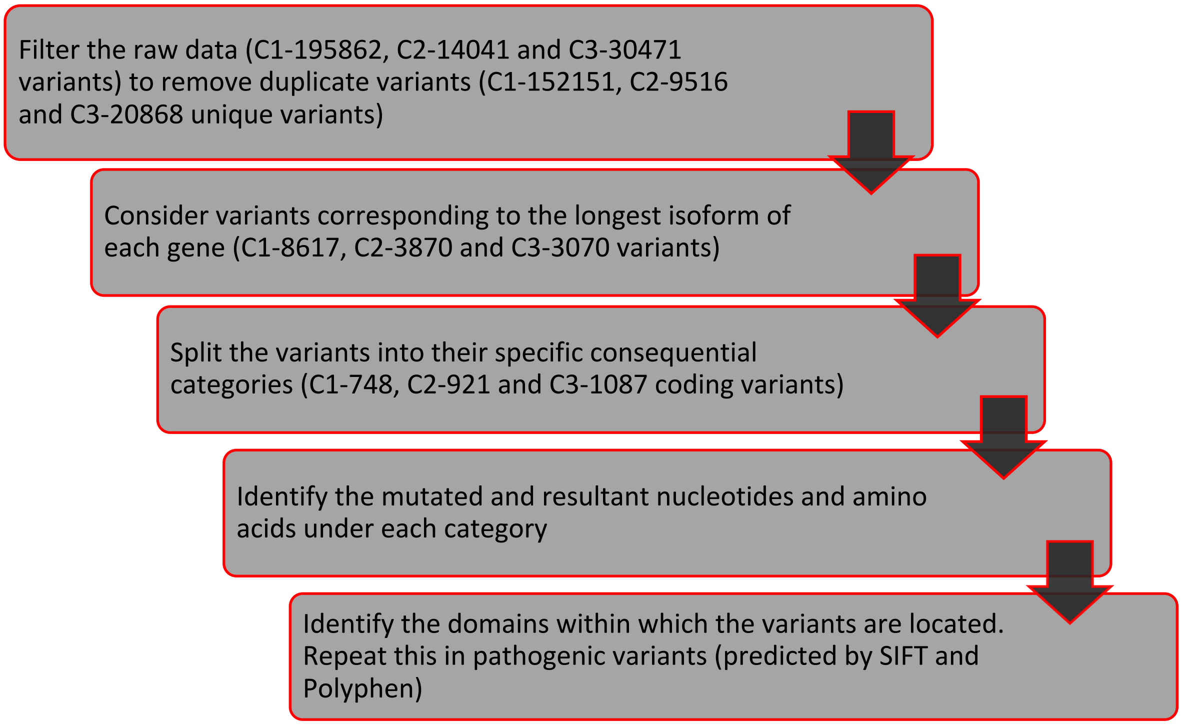

Supplementary Figure 2: Data flow diagram of raw data filtering and analysis techniques.

Numbers within parentheses represent the number of variants per gene; C1=*MYBPC1*, C2=*MYBPC2* and C3=*MYBPC3.*
